# Novel transcription regulatory sequences and factors of the immune evasion protein ICP47 (*US12*) of herpes simplex viruses

**DOI:** 10.1186/s12985-020-01365-3

**Published:** 2020-07-10

**Authors:** Jun-Ting Cheng, Ying-Ying Wang, Lin-Zhong Zhu, Ying Zhang, Wen-Qi Cai, Zi-Wen Han, Yang Zhou, Xian-Wang Wang, Xiao-Chun Peng, Ying Xiang, Hui-Yu Yang, Shu-Zhong Cui, Zhaowu Ma, Bing-Rong Liu, Hong-Wu Xin

**Affiliations:** 1 Laboratory of Oncology, School of Basic Medicine, Health Science Center, Yangtze University, 1 Nanhuan Road, Jingzhou, 434023 Hubei China; 2 Department of Biochemistry and Molecular Biology, School of Basic Medicine, Health Science Center, Yangtze University, Jingzhou, 434023 Hubei China; 3 Department of Interventional Therapy, Key laboratory of Carcinogenesis and Translational Research (Ministry of Education), Peking University Cancer Hospital & Institute. 52, Fucheng Road, Haidian District, Beijing, 100142 China; 4 Department of Laboratory Medicine, School of Basic Medicine, Health Science Center, Yangtze University, 1 Nanhuan Road, Jingzhou, 434023 Hubei China; 5 Department of Pathophysiology, School of Basic Medicine, Health Science Center, Yangtze University, Jingzhou, 434023 Hubei China; 6 Department of Gastroenterology, The First Affiliated Hospital of Zhengzhou University, Zhengzhou, 450052 China; 7 State Key Laboratory of Respiratory Disease, Affiliated Cancer Hospital Institute of Guangzhou Medical University, Guangzhou, 510095 China; 8 Lianjiang People’s Hospital, Guangdong, 524400 China

**Keywords:** HSV-1, HSV-2, *US12*, ICP47, Transcriptional regulation sequence (TRS), Transcriptional regulation factor (TRF)

## Abstract

**Background:**

Herpes simplex virus (HSV) can cause encephalitis. Its infected cell polypeptide 47 (ICP47), encoded by immediate-early gene *US12*, promotes immune escape. ICP47 was modified in the clinically approved oncolytic HSV (oHSV) T-Vec. However, transcription regulatory sequence (TRS) and transcription regulatory factor (TRF) of HSV *US12* are seldom reported.

**Methods:**

Previously, our laboratory isolated a new HSV strain named HSV-1-LXMW from a male patient with oral herpes in Beijing, China. Firstly, the genetic tree was used to analyze its genetic relationship. The *US12* TRS and TRF in HSV-1-LXMW were found by using predictive software. Secondly, the further verification by the multi-sequence comparative analysis shown that the upstream DNA sequence of HSV *US12* gene contained the conserved region. Finally, the results of literature search shown that the expression of transcription factors was related to the tissue affinity of HSV-1 and HSV-2, so as to increase the new understanding of the transcriptional regulation of HSV biology and oncolytic virus (OVs) therapy.

**Results:**

Here we reported the transcriptional regulation region sequence of our new HSV-1-LXMW, and its close relationship with HSV-1-CR38 and HSV-1-17. Importantly we identified eight different kinds of novel TRSs and TRFs of HSV *US12* for the first time, and found they are conserved among HSV-1 (c-Rel, Elk-1, Pax-4), HSV-2 (Oct-1, CF2-II, E74A, StuAp) or both HSVs (HNF-4). The TRFs c-Rel and Oct-1 are biologically functional respectively in immune escape and viral replication during HSV infection.

**Conclusions:**

Our findings have important implication to HSV biology, infection, immunity and oHSVs.

## Background

Tumors are heterogeneous and often resistant to chemotherapy and radiotherapy [1, 2], and no single treatment could be widespread applied or has full effectivity for cancer treatment [3–6]. OVs treatment are different from conventional chemotherapy and radiotherapy, and could provide additional treatment strategies [7]. Additionally, OVs are diverse in structure and biology, which spread among tumors with different kinetics and kill tumor cells through multiple mechanisms [8]. The oHSV T-VEC has been approved by FDA for patients with melanoma [9–11]. HSV, a member of the alpha-herpesviruses subfamily, which is an encapsulated DNA virus, offers particular benefits for use as a gene transfer vector, contains at least 120 kb of double-stranded DNA genome, encoding more than 70 genes [7, 12]. Type 1 and 2 HSVs (HSV-1 and HSV-2) are the most common and acute human pathogens. HSV-1 is normally related to oral-facial infections and may cause encephalitis in severe cases, while HSV-2 mostly induces genital infections and could cause mother-to-child transmission [13, 14].

HSV as an OVs has many favourable properties. Engineered oHSVs have been shown to be remarkably safe in clinical trials, and also have some evidence of their effectiveness [12]. Dlsptk, the first recombinant HSV, was generated by deleting the gene *UL23* encoding thymidine kinase (TK) [15]. The selectivity and efficacy of dlsptk established a principled proof for the application of HSV-1 genome deletions to carry out the tumor selectivity. However, from the standpoint of clinical application, the *UL23* deletion was eventually problematic for it causes dlsptk impervious to first-line anti-herpes pharmaceuticals, resulting in abandance of dlsptk. The second recombinant HSV, G207, is the first oHSV tested in clinical trials and it deletes the genes *UL39* (ICP6) and *RL1* (ICP34.5). As is well-known, ICP34.5, a key factor of HSV neurovirulence, can preclude the shut-off of protein synthesis in infected host cells. ICP6 is a determinant viral enzyme for HSV DNA synthesis, which is indispensable for virus replication in normal non-dividing cells [16]. Dlsptk and G207 are designed to weaken viral replication and reduce viral virulence in non-cancer cells. The third generation oHSV-1 vector, G47Δ, is based on G207 with additional ICP47 deletion, which surprisingly enhances viral replication and increases immune recognition of infected cells [17]. ICP47 deletion contains the promoter region of *US11* and also attenuates γ34.5 growth [18]. Importantly, because of ICP47 can block peptide loading of major histocompatibility complex I (MHC-I) molecules, G47∆ has induced an antitumor immune response for the ICP47 deletion. Therefore, increasing MHC I antigen presentation, stimulating cytotoxic lymphocytes and reducing NK cytolysis of infected cells can enhance anti-tumor immune response. Anti-tumor immune responses may be the key for the treatment of tumor metastasis .

ICP47, encoded by gene *US12*, is a polymorphous protein and could block RNA splicing in early infection, and then, shuttle viral mRNA from nucleus to cytoplasm in late infection [19]. ICP47 directly binds antigen-dependent transporter (TAP), limiting antigen trafficing, leading to the occurrence of empty MHC-I [20]. The functional domain of ICP47 has been mapped to 35 residues at the N-terminal, forming an extended helix-loop-helix structure in the lipid bilayer [20]. In addition, since ICP47 is too large to be easily transported by TAP, its high affinity binding traps TAP in an inactive conformation [21]. The binding of ICP47 stabilizes the inward conformation, and therefore blocks TAP from transitioning to the outward state in which the nucleotide binding domains (NBDs) form a closed dimer and the translocation pathway points to the endoplasmic reticulum (ER) cavity [22]. By blocking the entry of viral antigens into ER, HSV could avoid the attrack of cytotoxic T lymphocytes (CTLs), which may lead to immune escape of HSV and establish lifelong infection in the host cells. Interestingly, the ICP47 in G47Δ is deleted, and this keeps the cell surface MHC-I-antigen expression and allows to enhance antigen presentation [18]. Furthermore, G47∆ has been proved effective in animal tumor models of various cancers such as brain cancer, prostate cancer, breast cancer, schwannoma and human melanoma [23–25].

Currently, HSV *US12* is widely used in OVs modification, gene therapy and vaccine construction [25–27]. However, there are no reports on TRS and TRF of *US12* gene in HSV. As an immediate-early protein, its expression is regulated by the tri-partite Oct-1/HCF/VP16 complex [28, 29]. Identification of additional conserved promoter regulatory sequences that might further regulate its expression is certainly an important question. Here we sequenced the transcriptional regulation region of *US12* of our new HSV-1 strain LXMW, and for the first time identified novel TRS and TRF of HSV *US12*. These findings may have important impactions for HSV biology, infection, immunity and OVs.

## Methods

Previously, our laboratory isolated a new HSV strain named HSV-1-LXMW from a male patient with oral herpes in Beijing, China [30]. The detailed content of Cells, HSV-1 isolation and identification, and HSV genomic DNA sequencing analysis have been elaborated [30].

### Identification of the US12 potential transcriptional regulation region sequences in HSV

The online program NCBI (National Center for Biotechnology Information: https://www.ncbi.nlm.nih.gov/) was used to determine *US12* potential transcriptional regulation region sequences.

### Phylogenetic analysis of the transcriptional regulation region of the US12 gene

We used MEGA7 (https://www.megasoftware.net/), the application (APP), to analyze phylogenetic relationship.

### Prediction of US12 transcription regulatory sequences and factors

We used online program Match (http://gene-regulation.com/pub/programs.html) to predict the gene *US12* TRS and TRF according to their instruction.

### Alignment of the transcriptional regulation region sequences of US12

ApE (A plasmid Editor: http://biologylabs.utah.edu/jorgensen/wayned/ape/), the application (APP), was used to make the potential transcriptional regulation region sequences alignment of gene *US12* according to their manual.

## Results

### Identification of the US12 transcription regulatory region of HSV-1-LXMW

**Fig. 1:**
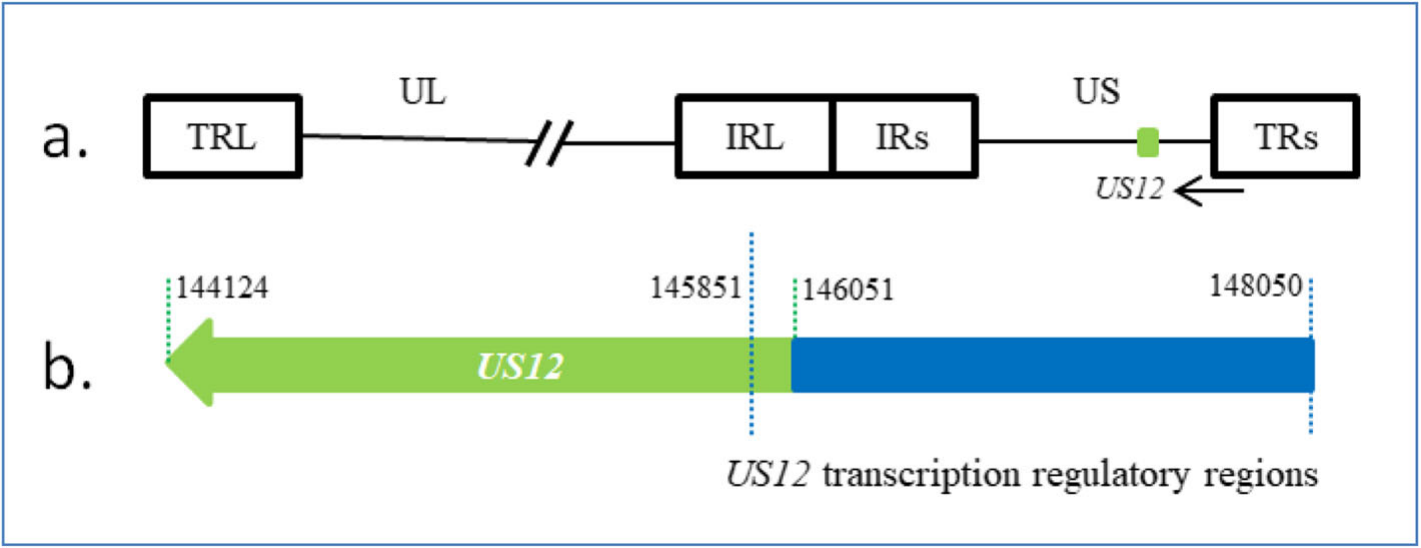
Transcription regulatory regions of *US12***.** a. Schematic of the HSV-1 genome showing the regions of *US12*. The HSV-1 genome consists of long and short unique regions (UL and US) each bounded by terminal (T) and internal (I) repeat regions (RL and RS). b. The DNA sequence of the US12 gene is marked in green and the transcription regulatory regions of *US12* is marked in blue

Using the nucleotide sequence database, we identified transcription regulatory regions of US12 (145851–148,050) as shown in Fig. 1. Please refer to supplementary material for the sequence of the transcription regulatory region. The transcription regulatory regions are 2000 bp upstream and 200 bp downstream of *US12* transcription initiation sites. Interestingly, the gene that encodes ICP47 is *US10*, not *US12* in HSV-2 strain HG52 and H1226. We summarized information about the HSV *US12* genomic DNA transcription regulatory regions of our new strain and 11 other strains studied in this article (Table 1).

**Table 1:** HSV *US12* genomic DNA sequencing

HSV Strain	Gene Bank ID	Tax-ID	Sub-Date	***US12*** transcription regulatory regions	University, Country
HSV-1 strain XLMW				145,851 / 148,050	Yangtze University, Jingzhou, China
HSV-1 strain 17	JN555585	10,299	2011-08-02	145,869 /148,068	RC University, Glasgow, UK
HSV-1 strain H129	GU734772	744,249	2010-01-18	145,769/ 147,968	Princeton University, USA
HSV-1 Strain CR38	HM585508	10,298	2013-10-17	145,315 / 147,514	MRC Virology Unit, UK
HSV-1 strain SC16	KX946970	10,309	2016-10-30	113,629/114,168	Severo Ochoa, Spain
HSV-1 strain KOS	JQ673480	10,306	2012-03-10	145,672 /147,871	University of Kansas, USA
HSV-1 strain Patton isolate	MF959544	10,308	2017-10-11	146,470 / 148,669	NYU, New York, USA
HSV-1 strain E19	HM585511	10,298	2013-10-22	144,988 / 147,187	University of Glasgow Centre for Virus Research, UK
HSV-1 strain F	GU734771	10,304	2010-01-18	145,795 / 147,994	Princeton University, USA
HSV-2 strain SD90e	KF781518	1,177,628	2013-10-25	147,503 / 149,702	Harvard Medical School, Boston, US
HSV-2 strain HG52	JN561323	10,310	2011-08-05	147,834 / 150,033	University of Glasgow, UK
HSV-2 strain H1226	KY922720	16,866	2017-09-27	147,094 /149,293	Pennslyvania State University, USA

### Phylogenetic analysis of HSV-1-LXMW and other 11 HSV strains

**Fig. 2:**
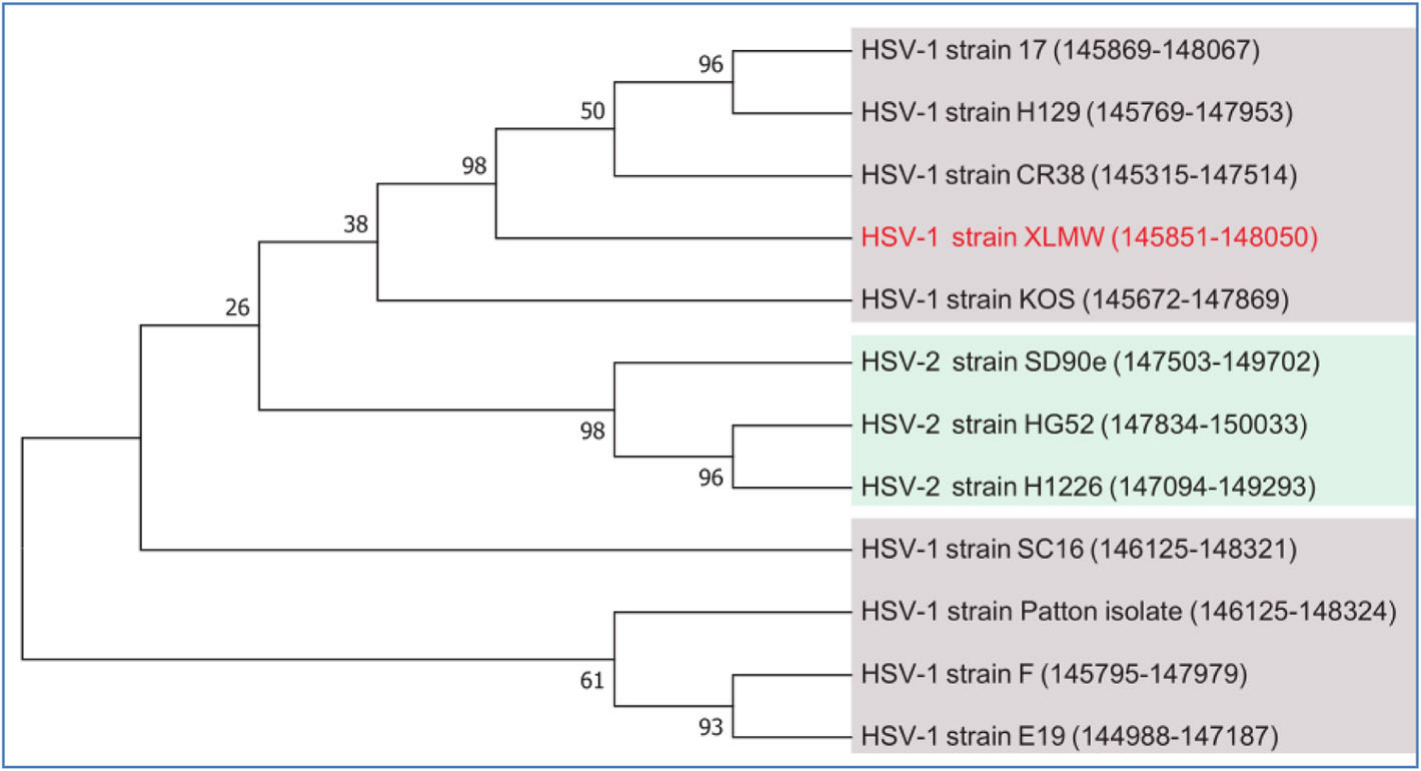
Phylogenetic analysis of HSV-1-LXMW together with 11 other HSV strains. Evolutionary analyses were conducted in MEGA7. The evolutionary history was inferred by using the Maximum Likelihood method based on the Tamura-Nei model. The bootstrap consensus tree inferred from 1000 replicates is taken to represent the evolutionary history of the taxa analyzed. Branches corresponding to partitions reproduced in less than 50% bootstrap replicates are collapsed

Based on the gene *US12* transcription regulatory region sequences in Table 1 of HSV-1-LXMW and other 11 HSV strains, including 8 HSV-1 strains (17, CR38, H129, SC16, KOS, Patton, E19 and F) and 3 HSV-2 strains (SD90e, HG52 and H1226), the phylogenetic analysis about the evolutionary relationship among HSV-1-LXMW and other 11 HSV strains were performed. The result shown high homology among our new strain HSV-1-LXMW and strains HSV-1-CR38, HSV-1-17 and HSV-1-H129. Our data again support that HSV-1-LXMW is a strain of HSV-1 (Fig. 2).

### Identification of the US12 TRS and TRF

Better understanding of *US12* transcriptional regulation is crucial for HSV biology and antitumor immune responses of oHSVs. Using Match, the online program, we find four major TRS of HSV-1-LXMW, which bind to c-Rel, HNF-4, ElK-1 and Pax-4, and three of HSV-1-17, which bind to c-Rel, HNF-4 and Pax-4. Interestingly, compared with the TRF of HSV-2, the difference between HSV-1 and HSV-2 is quite large. We find five major different kinds of TRSs of HSV-2-SD90e, which bind to HNF-4, CF2-II, E74A, Oct-1 and StuAp (Table 2).

**Table 2:** The *US12* TRS and TRF in HSVs

HSV strain	Matrix identifier	Position strand	Core match	Matrix match	Sequence	Factor name
**HSV-1 strain XLMW**	V$CREL_01	37 (+)	1.000	0.982	gggtcTTTCC	c-Rel
	V$HNF4_01	1020 (−)	0.883	0.898	ccctgtcCTTTTtcccacc	HNF-4
	V$ELK1_02	1875(+)	1.000	0.984	ggcgcCGGAAgccc	Elk-1
	V$PAX4_01	2029 (−)	0.888	0.833	gccacgggccgCTTCAcggcc	Pax-4
**HSV-1 strain 17**	V$CREL_01	37 (+)	1.000	0.982	gggtcTTTCC	c-Rel
	V$HNF4_01	1023 (−)	0.883	0.898	ccctgtcCTTTTtcccacc	HNF-4
	V$PAX4_01	2032 (−)	0.888	0.833	gccacgggccgCTTCAcggcc	Pax-4
**HSV-2 strain SD90e**	V$HNF4_01	746 (−)	1.000	0.928	gctcgcaCTTTGccctaat	HNF-4
	I$CF2II_01	767 (−)	1.000	1.000	tatATATAc	CF2-II
	V$HNF4_01	886 (−)	1.000	0.928	gctcgcaCTTTGccctaat	HNF-4
	I$CF2II_01	907 (−)	1.000	1.000	tatATATAc	CF2-II
	I$E74A_01	1076(+)	1.000	0.954	cgaaccCGGAAgggcag	E74A
	V$OCT1_Q6	1120 (−)	0.883	0.911	ctcaTTAGCatcgcg	Oct-1
	F$STUAP_01	1637 (−)	1.000	1.000	ggtCGCGAtg	StuAp

**Fig. 3:**
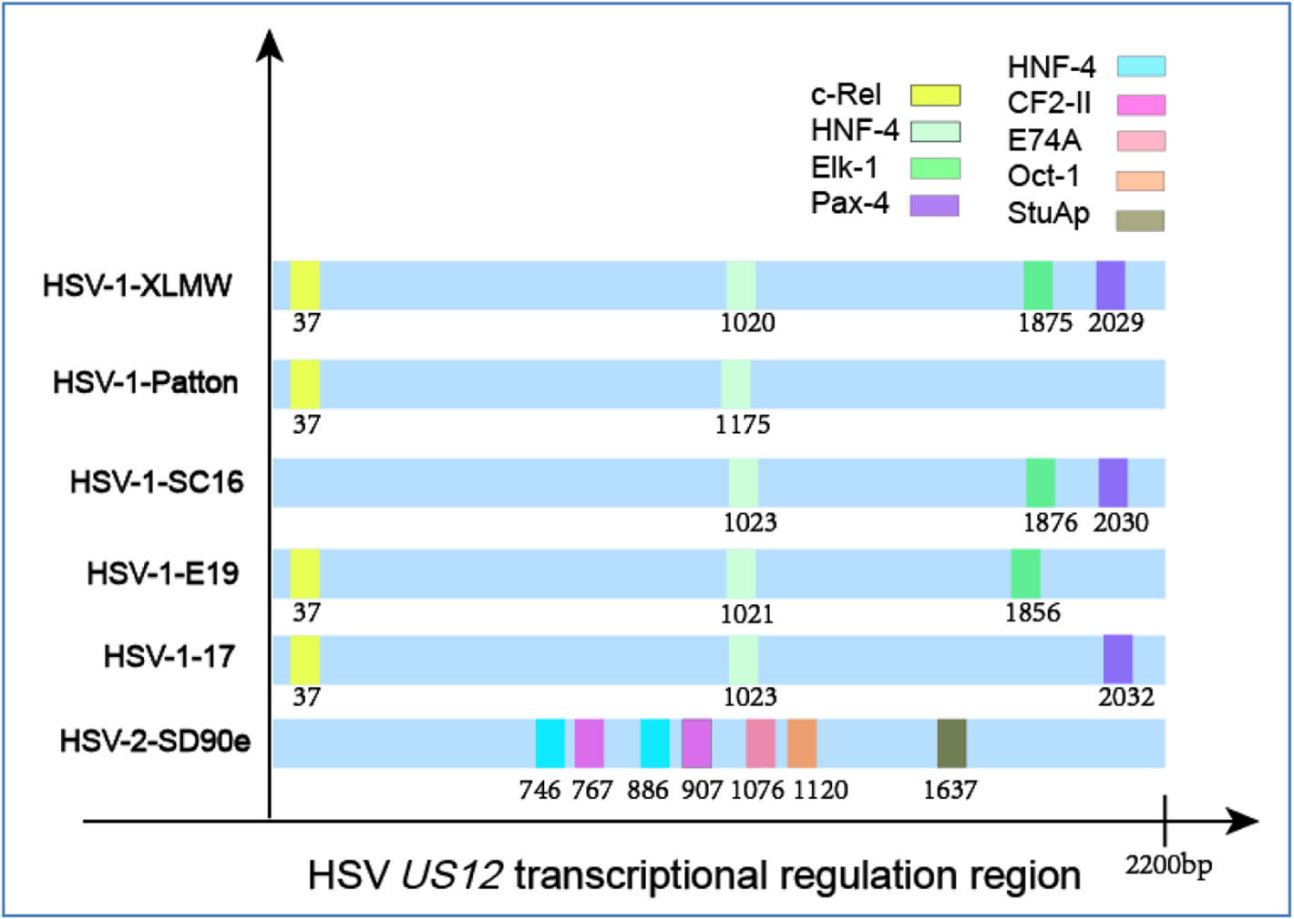
The *US12* TRSs and TRFs in HSVs. TRFs are represented in different colors, and the number represents the specific location of their binding TRFs

Further analysis of three more HSV-1 strains found that their TRS and TRF binding sites are similar with HSV-1-LXMW, but have minor differences (Fig. 3). Comparing to HSV-1-LXMW, there is no c-Rel binding site for HSV-1-SC16, no Elk-1 binding site for HSV-1-Patton and 17, and no Pax-4 binding site for HSV-1-Patton and E19. There is only one *US12* TRS binding to HNF-4 in all the HSV-1 strains analyzed, but there are two HNF-4 binding sites in HSV-2 strains.

### The TRS and TRF are conserved

**Fig. 4:**
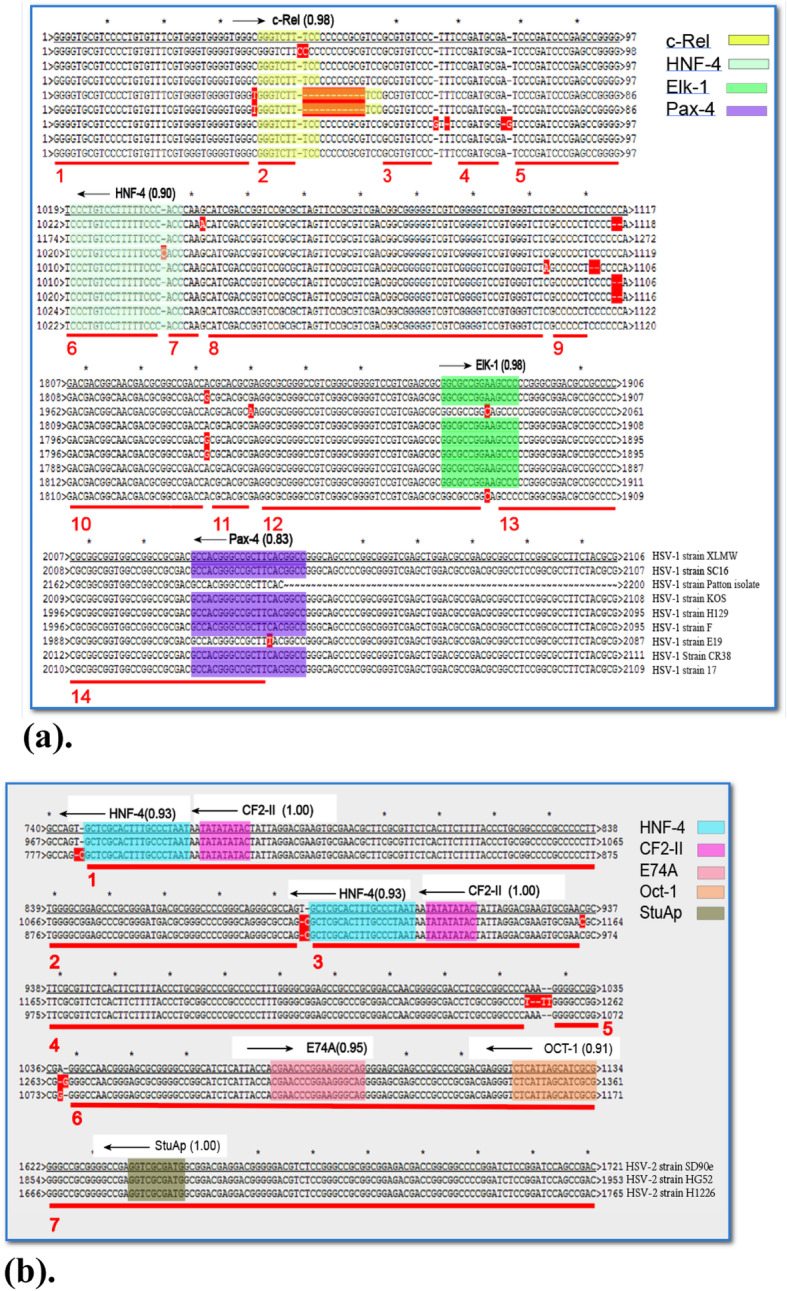
**a**. The *US12* TRSs and TRFs are conserved among HSV-1 strains. Red lines indicate the conserved regions 1–14. The TRSs and TRFs are shown in colored boxes. **b** The *US12* TRSs and TRFs are identical among HSV-2 strains. Red lines indicate the conserved regions 1–17. The TRSs and TRFs are shown in colored boxes

Conserved sequences refer to highly similar or identical nucleic acid sequences (RNA or DNA sequences), protein sequences, and their structures. Conserved sequences generally have functional value. Here, we found that the *US12* TRSs and TRF binding sites are conserved among the 9 HSV-1 and 3 HSV-2 strains (Fig. 4), indicating these conserved TRSs and TRFs are likely to be biologically functional.

Our multi-sequence alignment results indicated that in the *US12* transcriptional regulation regions there are less mutations among HSV-1 strains than mutations between HSV-1 and HSV-2. Between HSV-1-LXMW and HSV-1-172,184 base pairs are matched and only 5 base pairs are mismatched. However, there are only 468 base pairs matched between the HSV-1- LXMW and HSV-2-SD90e. Therefore, we decided to compare HSV type 1 and type 2 separately in ApE Program (Aligment parameters: Blocks: 10, mismatch penalty: 0, gap penalty: 0, gap Ext penalty: 0, everything else is at default). Results shown that our new strain HSV-1-LXMW was highly similar to HSV-1-17 and HSV-1-CR38 (Fig. 4a).

The alignment of the transcription regulatory region (nucleotides 1–2200) of HSV-1-XLMW strain to other 8 HSV-1 strains SC16, Patton, KOS, H129, F, E19, CR38 and 17, respectively, shown 2177, 2023, 2182, 2165, 2167, 2150, 2184 and 2184 matched base pairs, 11, 6, 6, 11, 11, 10, 3 and 5 mismatched base pairs, and 24, 324, 26, 48, 44, 80, 26 and 21 base pair gaps (Fig. 4a). The alignment of the transcription regulatory region (nucleotides 1–2200) of HSV-2- SD90e strain to other 2 HSV-2 strains HG52 and H1226, respectively, shown 1956 and 2128 matched base pairs, 3 and 2 mismatched base pairs, and 480 and 138 base pair gaps (Fig. 4b).

A sequence with five or more conserved base pairs is defined as a conservative region. From the alignment analysis, we found 14 conserved regions of 9 HSV-1 strains and 7 conserved regions of 3 HSV-2 strains (Fig. 4). The conserved regions of HSV-1-LXMW *US12* started respectively at 1, 37, 58, 70, 79, 1019, 1036, 1043, 1104, 1807, 1833, 1842, 1886, and 2007 base pairs. Interestingly, we found that most of the *US12* transcriptional regulation regions are overlapped with the TRSs identified above.

There are four TRFs in HSV-1-LXMW, KOS, H129, F and CR38 strains and three TRFs in HSV-1-SC16, E19 and 17 strains and two transcription factors in HSV-1-patton strain. Additionally, the TRFs binding sites of the HSV-1 strains are basically conserved (Fig. 4a). Interestingly, TRSs in the *US12* transcriptional regulatory region are identical in HSV-2 strains SD90e, HG52 and H1226 (Fig. 4b). Importantly, binding sites of c-Rel, HNF-4, Elk-1, Pax-4, CF2-II, E74A, Oct-1 or StuAp in *US12* transcriptional regulation regions are also conserved among HSV-1/2. HNF-4 is conserved in both HSV-1 and HSV-2 strains. Our findings support that the conserved TRSs and TRFs binding sites are closely linked with the gene *US12* functions. However, the TRSs and TRFs between HSV-1 and HSV-2 strains are quite different. Many bases are found unpaired in the sequence alignment, and that indicates different biological functions of these TRSs and TRFs in HSV-1 or HSV-2. Whether their functions exist in vivo or are related to the immune escape of HSV is not clear, thus, to validate these, further functional studies are needed.

### The TRFs c-Rel and Oct-1 are functional during HSV infection

To understand whether the identified conserved TRSs and TRFs are functional or are involved in the immune escape of HSV, we did a literature search of each of these TRSs and TRFs related to HSV-1 or HSV-2. We found that the TRFs of c-Rel and Oct-1 have been reported to be expressed and functional respectively in HSV-1 and HSV-1/2 infected cells (Table 3). These data are consistent with the identification of TRF c-Rel binding site in HSV-1 and Oct-1 binding site in HSV-2 (Fig. 3), supporting that these TRSs and TRFs are biologically functional.

**Table 3:** The TRFs c-Rel and Oct-1 are functional during HSV infection

Tissue type	HSV strain	Oct-1	c-Rel	Function	Ref.
Kidney: Vero cells	HSV-1 strain 17	–	c-Rel	As a novel cause of HSE disease susceptibility.	[31]
Hematological: Jurkat cells	HSV-1	–	p65/c-Rel	the p65/c-Rel heterodimer is responsible for the NF-kB-dependent induction of HIV-1 LTR in HSV- 1-infected cells.	[32]
Embryonic: WT and dOct MEF cells	HSV-1 strain F	Oct-1	–	Oct-1 is required for the formation of HSV replication factories and late gene expression.	[33]
Digestive: Hep2 cells	HSV-1 strain KOS	Oct-1	–	Oct-1 directly recognizes TAATGARAT elements in promoters of IE genes.	[34]
Urinary: COS-7 cells	HSV-1 strain KOS	Oct-1	–	Distinct conformations of Oct-1 on the BHV IE1 sites and on the HSV IE110 sites.	[35]
Genital: HeLa cells	HSV-1 strain F	Oct-1	–	late in infection Oct-1 is posttranslationally modified and exhibits a reduced capacity to bind to its cognate sites.	[36]
Genital: HeLa cells	HSV-1 strain KOS	Oct-1	–	Ser375 is important for the interaction of VP16 with Oct-1, and that the interaction is required to enable both proteins to bind to IE promoters.	[28]
Genital: HFF	HSV-1 strain KOS	Oct-1	–	forms a transactivation complex with the cellular proteins HCF-1 and HSV-1 VP16 tegument protein.	[29]
Genital: HeLa cells	HSV-2 strain 333	Oct-1	–	the HSV-2 protein forms a transcriptional complex with the cellular Oct-1 protein and target TAATGARAT elements from immediate-early promoters.	[37]

*HSE* Herpes simplex encephalitis, *HIV* human immunodeficiency virus, *LTR* long terminal repeat

It is well known that HSV-1 causes buccal ulcers and encephalitis. Interestingly, it’s reported that c-Rel is a novel cause of herpes simplex encephalitis susceptibility [38]. Studies have also shown that c-Rel is involved in immune evasion via interacting with viral nuclear protein *UL24* and endogenous NF-κB subunits p65 and p50, and inhibiting cGAS-STING mediated NF-κB promoter activity in HSV-1 infected cells. We would hypothesize that our newly identified HSV-1 specific c-Rel may bind to its *US12* TRSs, and activate *US12* (ICP47) expression in HSV-1 infected cells. In turn, ICP47 blocked HSV-1 antigen presentation, and promoted HSV-1 infection spread and herpes simplex encephalitis. Oct-1 activates IE-gene transcription through forming a transactivation complex with the cellular proteins HCF-1 and VP16 tegument protein in HSV-1 infected tissues [28, 29].

However, there was no report of *US12* transcriptional regulation by c-Rel or Oct-1, no report on c-Rel expression in HSV-2 infected tissues, and no report about expression and function of the other 6 identified TRFs of HNF-4, Elk-1, Pax-4, CF2-II, E74A and StuAp, and no report of any of the TRSs identified above in HSV.

### The HSV-1/2 tissue tropism and TRFs expression in different tissues

Tissue tropism is the cells and tissues of a host that support the growth of a particular virus or bacterium. Some bacteria and viruses have a wide range of tissue tropism and can infect many types of cells and tissues, while other viruses may infect mainly individual tissues. Here we summarized the HSV-1/2 tissue tropism and the TRFs expression in different tissues (Table 4). According to the results, HSV-1 specific c-Rel, Elk-1, and Pax-4 are highly expressed in tissues above the abdomen, including oral cavity, tongue and head, and Oct-1, HNF-4 and CF2-II are highly expressed in tissues within the genital system. c-Rel belongs to the nuclear factor κB (NF-κB) family, and plays a crucial role in mammalian B and T cell function [39]. Elk-1 is involved in ERK-induced cellular proliferation, and its transcriptional activity is regulated by ubiquitination at lysine 35 (K35) [40]. Pax proteins are crucial in stem cell biology and organ development. Pax-4 is known to be a major regulator of pancreatic cell development and differentiation, and its transactivation domain was localized within its C-terminal region [41]. OCT-1 (Pou2f1) is well known as a widely expressed TRFs in most cells and tissues. Recently, a series of studies have reported that OCT-1 plays a critical role in CD4^+^ T cell function through mediating long-range chromosomal interactions and regulating gene expression during differentiation [42]. Hepatocyte nuclear factor 4 (HNF-4) is enriched in liver extracts and belongs to the steroid hormone receptor superfamily [43]. C(2)-H(2)-type zinc-finger transcription factor II (CF2-II) may potentially regulates diverse sets of target genes during cell development and the CF2-II recognition properties depends largely on the COOH-terminal DNA binding domain [44]. E74A belongs to Ets transcription protein, which is involved in multifarious important biological processes. Study has demonstrated that ecdysone inducible TRFs E74A could directly regulate the EO gene expression in silkworm [45]. StuAp is a member of fungal TRFs family that regulates cell cycle progression or development. Further, StuAp belongs to a sub-family possessing the conserved APSES domain. Study has shown that StuAp acts as a transcriptional repressor in A.nidulans, but as a weak activator in budding yeast [46].

**Table 4:** The HSV-1/2 tissue tropism and the TRFs expression in different tissues *H* high-expression, *M* middle expression, *L* little expression, *N* no-expression; The result from: http://biogps.org. Grading was based on fold increases compared to median fluorescence intensity on Affymetrix microarray chips at 0–2.5 (L), > 2.5- < 5 (M), > 5 (H)

System	Cell/ tissue	HSV-1	HSV-2	c-Rel	HNF-4	Elk-1	Pax-4	CF2-II	OCT-1
**Blood system**	CD34^+^ stem cell	+	_	H	H	H	H	H	H
	721 B lymphoblasts	+	_	H	H	H	H	M	H
	CD19 + B cell	+	_	H	H	H	H	M	H
	Leukemia lymphoblastic	+	_	M	M	M	M	L	L
	Bonemarrow	+	_	H	M	H	H	M	H
	Pituitariy	+	_	H	H	H	H	H	M
**Head**	Prefrontal Cortex	+	_	H	H	H	H	H	H
	Pineal	+	_	H	H	H	H	H	M
	Tongue	+	_	H	M	H	H	L	M
	Tonsil	+	_	H	M	M	H	L	L
	Retina	+	_	H	H	H	H	H	M
	Trigeminal ganglion	+	_	M	H	H	H	L	L
	Cerebellum	+	_	H	H	M	H	H	M
**Viscera**	Heart	+	_	H	H	H	H	H	M
	Lung	+	_	H	H	H	H	H	M
	Liver	+	_	H	H	H	H	H	M
	Kidney	+	_	M	M	M	M	H	L
	Smooth Muscles	+	_	H	H	H	H	H	M
	Adipocyte	+	_	H	M	M	H	L	L
**Secretory system**	Adrenalgland	+	_	M	M	H	H	L	L
	Pancreaticlstet	+	_	H	H	H	H	H	M
**Genital system**	Placenta	+	+	H	H	H	M	H	M
	Fetalthyroid	+	+	H	M	M	M	H	M
	Uterus	+	+	M	M	M	M	M	L
	Testis	+	+	M	M	M	M	H	L
	Ovary	+	+	M	M	L	L	L	M

### The US12 transcription and ICP47 function during the HSV infection process

**Fig. 5:**
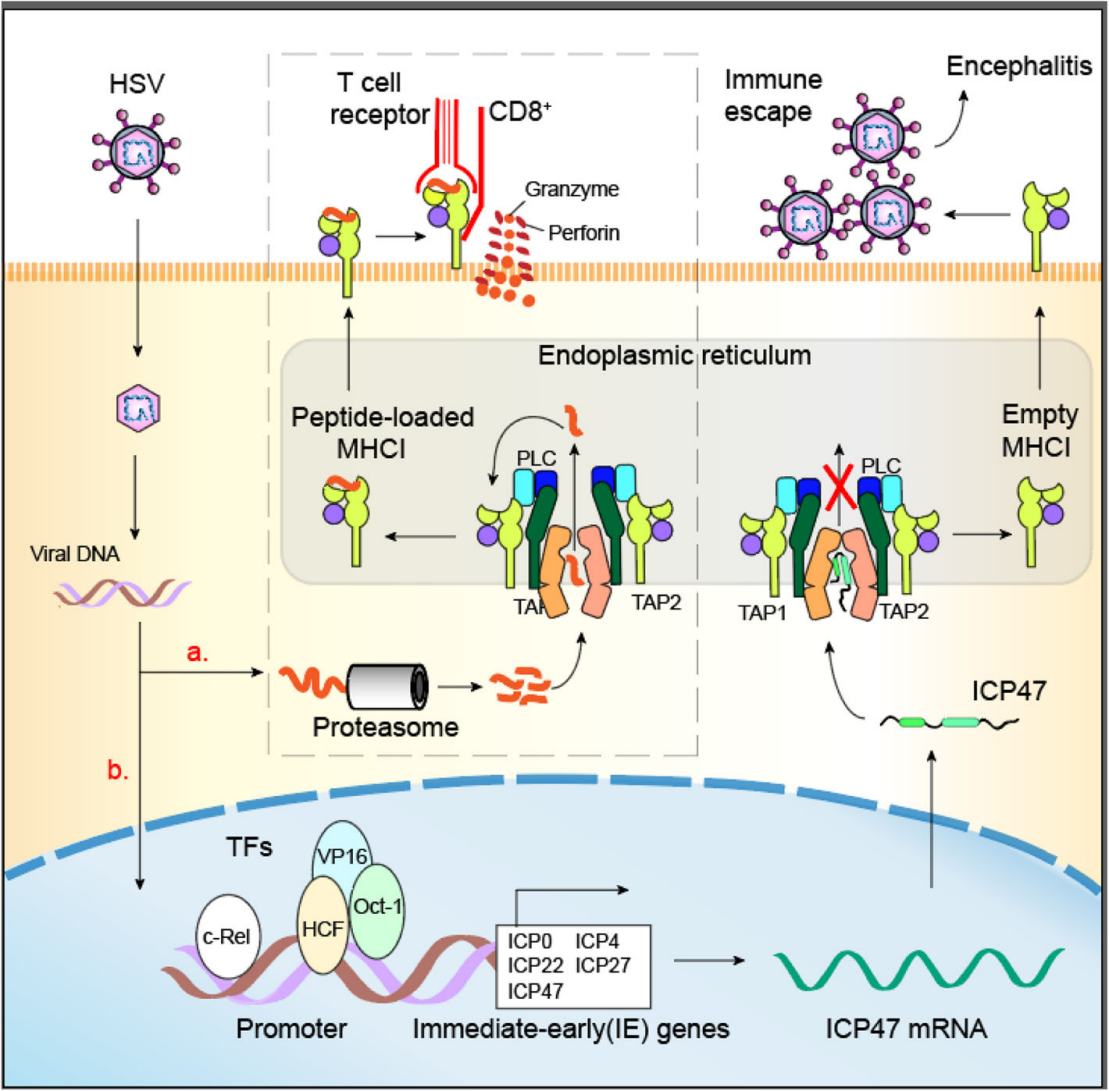
ICP47 function during HSV infection. a Intracellular antigenic peptides, mainly generated by the proteasome, are transported into the endoplasmic reticulum by the TAP and then loaded onto the nascent MHC I molecules, which are exported to the cell surface and present peptides to the immune cells. Cytotoxic T lymphocytes recognize and kill the infected cells by granzyme and perforin. b ICP47 can preclude peptide binding and traps TAP in an inward-facing conformation. Binding of ICP47 stabilizes the inward-facing conformation, and thus prevents TAP from transitioning to an outward-facing state, resulting in the emergence of empty carrier MHC I molecules. Therefore, CD8^+^ T cells could not recognize them, and HSVs could avoid the immune responses. We hypothesize that c-Rel bind to its *US12* TRS, and enhance *US12* (ICP47) expression, leading to HSV-1 immune evasion and HSV-1 encephalitis

To better understand the significance of the newly identified *US12* transcriptional regulation, we summarize the *US12* transcription and ICP47 function during the HSV infection process (Fig. 5). TAP plays a crucial role in MHC I antigen presentation and has become an important target for viral immune escape strategies. In the long-term process of virus-host co-evolution, herpes viruses independently obtained an efficient way to block TAP-mediated peptide transport via the viral immune evasion protein ICP47, which blocks the binding of peptide to TAP by capturing TAP in the endogenous conformation [27]. Interestingly, in our study, we found that two crucial TRFs, c-Rel and Oct-1, play a variety of roles in the growth, proliferation, and survival of mature T cells, which might associate with the viral immune evasion via HSV ICP47. Studies have shown that HSV-1 have evolved complex mechanisms to disrupt the antiviral response via affecting the NF-κB. For example, in HSV-1, ICP0 interacts with p65 and p50 and then degrades p50 through regulating E3 ubiquitin ligase activity [47]. Protein kinase *US3* was shown to inhibit NF-κB activity via making p65 hyperphosphorylation at serine 75 and blocking its nuclear translocation [48]. Besides, ICP27 blocks the phosphorylation of IκB to inhibit NF-κB activation. Furthermore, our data also shown that c-Rel is conserved in HSV-1, which inhibits NF-κB promoter activity. Importantly, Oct-1 plays a key role in CD4 T cells, mediating long-range chromosomal interactions and differentiation through regulating gene expression, and has a critical protection effect on viruses and pathogens [42] and further, Oct-1 also is conserved in HSV-2.

## Discussion

HSV ICP47 can bind TAP and block antigen presentation. Transcriptional regulation of *US12* is important for ICP47 functioning. However, TRSs and TRFs of HSV *US12* are seldom reported. In this study, we reported the transcriptional regulation region sequence of our newly isolated strain HSV-1-LXMW in China, and found it is closely related to HSV-1-CR38 and HSV-1-17 in UK. We identified eight different kinds of novel TRSs and TRFs of HSV *US12* for the first time. These identified TRSs and TRFs are conserved among HSV-1 (c-Rel, Elk-1, Pax-4), HSV-2 (Oct-1, CF2-II, E74A, StuAp) or both of them (HNF-4). Two of the TRFs c-Rel and Oct-1 are biologically functional in vitro respectively in immune escape and viral replication during HSV infection. We further hypothesize a novel mechanism of HSV-1 encephalitis by c-Rel activated ICP47-mediated immune escape. These findings may have important implication to our understanding of HSV biology, infection, immunity and OVs.

oHSV-1 has become one of the most promising OVs at present [49]. In 2015, talimogene laherparepvec (T-VEC), a kind of oHSV, was approved by FDA for the treatment of advanced melanoma [50–52]. In T-VEC, ICP47 was deleted to prevent limitation of viral antigen presentation, and increase the *US11* gene expression, and virus replication in cancer cells without reducing tumor selectivity [53]. Considering that the immune escape function of ICP47, the construction of gene therapy vectors precede a new perspective. For instance, Adeno-associated virus gene therapy of Duchenne muscular dystrophy was achieved by expression ICP47 [54]. Additionally, study has also reported another recombinant adenovirus vector expressing ICP47 protein to reduce the stimulation of dendritic cells [55].

Future functional studies of these novel TRSs and TRFs, and their roles in HSV replication, infection, immunity, tissue tropism, encephalitis and OVs are warranted.

## Conclusions

We identified eight different kinds of novel TRFs and TRFs of HSV *US12* for the first time, and found they are conserved among HSV-1 (c-Rel, Elk-1, Pax-4), HSV-2 (Oct-1, CF2-II, E74A, StuAp) or both HSVs (HNF-4). The c-Rel and Oct-1 are biologically functional respectively in immune escape and viral replication during HSV infection. We further hypothesized a novel mechanism of HSV-1 encephalitis caused by c-Rel activated ICP47-mediated immune escape.

## Supplementary information

**Additional file 1.** Using the nucleotide sequence database, we identified transcription regulatory regions of US12 (145851–148,050). The transcription regulatory regions are 2000 bp upstream and 200 bp downstream of US12 transcription initiation sites.

## Data Availability

All data from the current study are available from the corresponding author on request.

## Abbreviations

HSV: Herpes simplex virus
ICP47: Infected cell polypeptide 47
oHSV: oncolytic HSV
TRSs: Transcription regulatory sequences
TRFs: Transcription regulatory factors
OVs: Oncolytic viruses
TK: Thymidine kinase
MHC-I: Major histocompatibility complex I
TAP: Transporter associated with antigen processing
NBDs: Nucleotide binding domains
ER: Endoplasmic reticulum
CTLs: Cytotoxic T lymphocytes
NF-κB: Nuclear factor κB
HNF-4: Hepatocyte nuclear factor 4
CF2-II: C(2)-H(2)-type zinc-finger transcription factor II
T-VEC: Talimogene laherparepvec
