# Supplementary material for: Novel transcription regulatory sequences and factors of the immune evasion protein ICP47 (US12) of herpes simplex viruses
Source: Virol J. 2020 Jul 10;17:101. doi: 10.1186/s12985-020-01365-3 (PMC7377220; doi:10.1186/s12985-020-01365-3)
Supplement: Supplementary file 1 — Additional file 1. Using the nucleotide sequence database, we identified transcription regulatory regions of US12 (145851–148,050). The transcription regulatory regions are 2000 bp upstream and 200 bp downstream of US12 transcription initiation sites. [file 12985_2020_1365_MOESM1_ESM.docx]

**Supplementary Materials**

The sequence of the transcription regulatory region of our recently isolated strain HSV-1-XMW is shown here (145851- GGGGTGCGTCCCCTGTGTTTCGTGGGTGGGGTGGGCGGGTCTTTCCCCCCCGCGTCCGCGTGTCCCTTTCCGATGCGATCCCGATCCCGAGCCGGGGCGTCGCGATGCCGACGCCGTCCGCTCCGACGGCCCTCTGCGACTCCCGCTCCCGGTCCGCGTGCTCCGCAGCCGCTCCCGTCGTTCGTGGCCGGCGCCGTCTGCGGGCGTCGGTCGCGCCGGGCCTTTATGTGCGCCGGAGAGACCCGCCCCCCGCCGCCCGGGCCCGCCCCCGGGGCGGCGCGGAGTCGGGCACGGCGCCAGTGCTCGCACTTCGCCCTAATAATATATATATATTGGGACGAAGTGCGAACGCTTCGCGTTCTCACTTCTTTTACCCGGCGGCCCCGCCCCCTTGGGGCGGTCCCGCCCGCCGGCCAATGGGGGGGCGGCAAGGCGGGCGGCCCTTGGGCCGCCCGCCGTCCCGTTGGTCCCGGCGTCCGGCGGGCGGGACCGGGGGCCCGGGGACGGCCAACGGGCGCGCGGGGCTCGTATCTCATTACCGCCGAACCGGGAAGTCGGGGCCCGGGCCCCGCCCCCGGCCCGTTCCTCGTTAGCATGCGGAACGGAAGCGGAAACCGCCGGATCGGGCGGTAATGAGATGCCATGCGGGGCGGGGCGCGGACCCACCCGCCCTCGCGCCCCGCCCNNNNNNNNTGGCGCGGATGGGCGGGGCCGGGGTTCGACCAACGGGCCGCGGCCACGGGCCCCCGGCGTGCCGGCGTCGGGGCGGGGTCGTGCATAATGGAATTCCGTTCGGGGTGGGCCCGCCGGGGGGGCGGGGGGCCGGCGGCCTCCGCTGCTCCTCCTTCCCGCCGGCCCCTGGGACTATATGAGCCCGAGGACGCCCCGATCGTCCACACGGAGCGCGGCTGCCGACACGGATCCACGACCCGACGCGGGACCGCCAGAGACAGACCGTCAGACGCTCGCCGCGCCGGGACGCCGATACGCGGACGAAGCGCGGGAGGGGGATCGGCCGTCCCTGTCCTTTTTCCCACCCAAGCATCGACCGGTCCGCGCTAGTTCCGCGTCGACGGCGGGGGTCGTCGGGGTCCGTGGGTCTCGCCCCCTCCCCCCATCGAGAGTCCGTAGGTGACCTACCGTGCTACGTCCGCCGTCGCAGCCGTATCCCCGGAGGATCGCCCCGCATCGGCGATGGCGTCGGAGAACAAGCAGCGCCCCGGCTCCCCGGGCCCCACCGACGGGCCGCCGCCCACCCCGAGCCCAGACCGCGACGAGCGGGGGGCCCTCGGGTGGGGCGCGGAGACGGAGGAGGGCGGGGACGACCCCGACCACGACCCCGACCACCCCCACGACCTCGACGACGCCCGGCGGGACGGGAGGGCCCCCGCGGCGGGCACCGACGCCGGCGAGGACGCCGGGGACGCCGTCTCGCCGCGACAGCTGGCTCTGCTGGCCTCCATGGTAGAGGAGGCCGTCCGGACGATCCCGACGCCCGACCCCGCGGCCTCGCCGCCCCGGACCCCCGCCTTTCGAGCCGACGACGATGACGGGGACGAGTACGACGACGCAGCCGACGCCGCCGGCGACCGGGCCCCGGCCCGGGGCCGCGAACGGGAGGCCCCGCTACGCGGCGCGTATCCGGACCCCACGGACCGCCTGTCGCCGCGCCCGCCGGCCCAGCCGCCGCGGAGACGTCGTCACGGCCGGCGGCGGCCATCGGCGTCATCGACCTCGTCGGACTCCGGGTCCTCGTCCTCGTCGTCCGCATCCTCTTCGTCCTCGTCGTCCGACGAGGACGAGGACGACGACGGCAACGACGCGGCCGACCACGCACGCGAGGCGCGGGCCGTCGGGCGGGGTCCGTCGAGCGCGGCGCCGGAAGCCCCCGGGCGGACGCCGCCCCCGCCCGGGCCATCCCCCCTCTCCGAGGCCGCGCCCAAGCCCCGGGCGGCGGCGAGGACCCCCGCGGCCTCCGCGGGCCGCATCGAGCGCCGCCGGGCCCGCGCGGCGGTGGCCGGCCGCGACGCCACGGGCCGCTTCACGGCCGGGCAGCCCCGGCGGGTCGAGCTGGACGCCGACGCGGCCTCCGGCGCCTTCTACGCGCGCTATCGCGACGGGTACGTCAGCGGGGAGCCGTGGCCCGGCGCCGGGCCCCCGCCCCCGGGGCGGGTGCTGTACGGCGGCCTGGGCGACAGCC -148050).

**Supplementary Materials Legends**

Using the nucleotide sequence database, we identified transcription regulatory regions of US12 (145851-148050). The transcription regulatory regions are 2000bp upstream and 200bp downstream of US12 transcription initiation sites.
